# Relationships of trait anxiety, test anxiety, and academic performance of Chinese undergraduates with typical developments and high- and typical-functioning specific learning disabilities

**DOI:** 10.1007/s11881-025-00328-z

**Published:** 2025-04-16

**Authors:** Li-Chih Wang, Kevin Kien-Hoa Chung

**Affiliations:** 1 Department of Special Education and Counselling, The Education University of Hong Kong, 10 Lo Ping Road, Tai Po, Hong Kong, N.T China; 2 Department of Early Childhood Education, The Education University of Hong Kong, 10 Lo Ping Road, Tai Po, Hong Kong, N.T China

**Keywords:** Trait anxiety; Test anxiety; Specific learning disabilities; Chinese undergraduates

## Abstract

This study aimed to compare trait anxiety and test anxiety among Chinese undergraduates with typical development, high-functioning specific learning disabilities (SLD), and typical-functioning SLD, and to examine the mediating role of test anxiety in the relationship between trait anxiety and academic performance across these three groups. The study included 239 s-year undergraduate students from universities in Taiwan, with 134 typically developing students, 54 students with typical-functioning SLD, and 51 students with high-functioning SLD. Our results indicated that the high-functioning SLD group reported significantly lower levels of trait anxiety and test anxiety compared to both the typically developing and typical-functioning SLD groups. The typical-functioning SLD group exhibited the highest levels of test anxiety. Mediation analyses revealed that test anxiety significantly mediated the relationship between trait anxiety and academic performance in the high-functioning SLD group, while only a direct effect of trait anxiety on academic performance was found in the typical-functioning SLD group. No significant direct or indirect effects were found in the typically developing group. Our findings highlight distinct anxiety profiles and differential patterns of direct and indirect effects of trait anxiety on academic performance among Chinese undergraduates with high-functioning SLD, typical-functioning SLD, and typical development.

## Introduction

Specific learning disabilities (SLD) is one of the most common neurodevelopmental disorders, and it is estimated to have around 5% prevalence (American Psychiatric Association, DSM-5 Task Force, 2013), characterized by impairments in academic achievement despite adequate cognitive and learning opportunities (Peterson et al., 2021). Diagnosis has centered on school-aged children demonstrating achievement gaps. However, accumulating evidence indicates those with SLD also frequently experience significant socioemotional challenges (Wang, 2021), which can critically impede their learning effectiveness (Gerber, 2012). Understanding the features of students with SLD across different ages, including adulthood, is crucial, as support needs evolve over time. Of particular relevance, several studies reveal adults retaining SLD self-report chronically elevated anxiety vulnerability relative to typically developing peers (Carawan et al., 2016; Nelson & Harwood, 2011; Wang & Chung, 2022; Wang et al., 2021).

According to regulatory focus theory, the continued exertion of compensatory effort toward remediating one’s cognitive deficiencies paired with academic struggles solidifies internalized failure orientations and broad avoidance motivation for such students over time (Scholer & Higgins, 2013). Thereby, the temporary worries and uncertainty that emerge from early classroom learning contexts may gradually generalize across development, commonly manifesting as chronic anxiety conditions during adulthood among those who retain functional SLD barriers (Carawan et al., 2016). This affective trajectory is likely exacerbated by the abrupt loss of specialized external assistance and accommodations in high school and college.

Thus, in the absence of transition support infrastructure to facilitate proving or concealing evolving SLD needs amid new environments, socioemotional disturbances tied to a neurocognitive disorder originating in childhood persist and likely compound unattended across adulthood. Particularly, the higher level of anxiety of individuals with SLD, especially for adults with SLD, has been repeatedly reported, and such a phenomenon happens in various aspects of anxiety. One of them is trait anxiety (e.g., Carroll & Iles, 2006; Hoy et al., 1997).

### Trait anxiety of students with SLD

Trait anxiety is a stable personality tendency reflecting chronic individual differences in perceiving situations as threatening and experiencing anxious reactions (Spielberger, 1972). This construct has been extensively studied among individuals with SLD, as it is believed to play a significant role in their emotional and academic experiences. Research has consistently demonstrated that children and adolescents with SLD report substantially higher levels of trait anxiety compared to their typically developing peers (Nelson & Harwood, 2011; Peleg, 2009). These findings suggest that individuals with SLD are more prone to perceiving various situations as threatening and responding with heightened anxiety, which may contribute to the challenges they face in academic and social contexts.

An extensive research literature has delineated affective functioning profiles among students diagnosed with SLD (Nelson et al., 2007), with a predominant focus on trait anxiety. Findings overwhelmingly demonstrate that children and adolescents with SLDs self-report substantially higher general trait anxiety compared to typically developing peers (Nelson & Harwood, 2011). For example, studies employing anxiety inventories reveal nearly twofold increases in trait anxiety scale scores for SLD groups relative to typically developing matched controls (Peleg, 2009). Such anxiety vulnerability has been evidenced across the age spectrum in youth with diagnosed SLDs (Grills-Tacquechel et al., 2012).

Cognitive interference theoretical frameworks posit that the constant exertion and compensatory efforts needed by those with SLD to mitigate their inherent aptitude-achievement discrepancies may predispose chronic worries and instability in self-concept over time (Eysenck et al., 2007; Owens et al., 2012). The strain imposed by ongoing struggles to master basic academic skills well enough to minimize negative feedback (Scholer & Higgins, 2013), alongside stigma surrounding special assistance needs, makes perceived coping ability chronically uncertain for this population. Thereby, SLD confers trait-based anxiety risks beginning as early as initial school enrollment through adulthood (Peleg, 2009). However, most of the current studies are dedicated to investigating the levels of those with SLD, compared to their peers, but less focused on the impact of trait anxiety on the academic performance of those with SLD.

### The relationship between trait anxiety and academic performance

In recent years, trait anxiety has been frequently studied in relation to academic performance. Specifically, research has aimed to understand how trait anxiety levels impact students’ reading comprehension and other literacy skills, but there have been inconsistent results.

A number of studies have clearly shown that students with higher levels of trait anxiety display poorer performance on reading assessments, including measures of reading fluency, reading comprehension, and general literary competency (Suárez-Pellicioni et al., 2016; Tysinger et al., 2010). Tysinger et al. (2010) administered standardized reading fluency and comprehension tests to middle school students, finding that those rating higher in self-reported trait anxiety had lower average scores. Additionally, Suárez-Pellicioni et al. (2016) had university students complete verbal reasoning and reading comprehension tests, demonstrating that participants with anxiety disorders and presumably elevated trait anxiety performed significantly worse than their non-anxious peers. The weaker literary abilities displayed by highly trait-anxious students align with prominent cognitive interference theories proposing that anxiety detracts attention and working memory capabilities necessary for handling complex academic material (Eysenck et al., 2007). In other words, worry and intrusive thoughts triggered by trait anxiety are believed to compete for phonological short-term memory resources vital for reading and verbal processing.

On the contrary, a number of researchers have failed to detect any meaningful correlations between trait anxiety levels and students’ scores on standardized reading tests or other measures of literary competency (Lee, 1999; Sylvia et al., 2023). For example, Sylvia et al. (2023) tested the cognitive processes, including trait anxiety and fluid reasoning, through which test anxiety may affect reading comprehension performance, and no significant contribution was found from trait anxiety. Although it may seem counterintuitive, a few studies have even evidenced that students classified as highly trait-anxious surpassed their less anxious peers on certain reading exam questions or tasks requiring close passage analysis (Ackerman & Heggestad, 1997).

Understanding how trait anxiety impacts academic performance is crucial, especially for students with SLD who may already face academic challenges. Anxiety can interfere with cognitive processes essential for learning, such as attention and working memory (Eysenck et al., 2007). By investigating this relationship, we can identify factors that exacerbate academic difficulties and develop interventions to support students with SLD.

### The necessity to consider test anxiety as a potential mediator

To reconcile these inconsistencies, Everson et al. (1991) propose that the trait anxiety-reading ability relationship likely depends on a third variable—the extent to which a given reading assessment also elicits state test anxiety.

Test anxiety refers to the specific emotional, cognitive, and physiological responses triggered by evaluative situations such as exams (Cassady, 2002; Spielberger & Vagg, 1995). In contrast to trait anxiety, which is conceptualized as a relatively stable disposition to perceive a broad range of circumstances as threatening (Spielberger, 1972), test anxiety is more narrowly focused on performance outcomes in testing contexts. Consequently, individuals with elevated trait anxiety generally exhibit a lower threshold for experiencing state anxiety during exams, increasing their vulnerability to test anxiety (Lowe et al., 2008). This situationally driven distress may act as a key mechanism by which trait anxiety undermines academic performance, especially in reading and other domains where evaluative tasks play a central role (Everson et al., 1991; Lee, 1999).

High-stakes standardized tests appear most likely to spur debilitating evaluative stress and corresponding working memory deficits for students already prone to anxiety (Luethi et al., 2009). Thus, while trait anxiety alone may not reliably predict reading outcomes, the combination of both chronic anxiety traits and situation-specific testing anxiety seems to substantially undermine literacy skill demonstration. Elucidating the precise effects then relies on incorporating measurements of test anxiety into study designs, which few studies have attempted. Ultimately, contradictory evidence underscores the need to better characterize how general tendencies toward anxiety interact with context-dependent evaluation stressors to impact students’ ability to comprehend and analyze text passages.

In other words, these contradictory findings suggest that the relationship between trait anxiety and reading performance depends on moderating variables that have been overlooked. One potential explanation lies in test anxiety, which refers to anxiety experienced in response to evaluations and examinations. Students higher in both trait and test anxiety tend to show worse reading outcomes compared to students with either anxiety type alone (Lee, 1999). This pattern aligns with processing efficiency theory (Eysenck & Calvo, 1992), which proposes that anxiety impairs processing and short-term memory capacities vital for complex cognitive tasks. Trait anxiety chronically undermines these resources while test anxiety taxes them further in evaluative settings. Together, they restrict working memory even more, compromising literacy demonstrations.

Theoretical models posit that trait anxiety predisposes individuals to experience test anxiety (Spielberger & Vagg, 1995), and it argues that the mechanism relates to trait anxiety lowering the threshold at which exam/testing situations provoke anxiety. High trait anxiety essentially acts as a predisposition/risk factor for interpreting testing as threatening and reacting with heightened test anxiety (Lowe et al., 2008). Those with elevated trait anxiety levels are thought to have a lower threshold for anxiety arousal in evaluative situations like exams (Lowe et al., 2008). However, test anxiety is conceptualized as distinct from trait anxiety in that situational factors related to testing also contribute to the experience of anxiety during exams (Cassady, 2002).

This proposes that high test anxiety may explain cases where high trait anxiety associates with poorer reading, whereas trait anxiety alone shows little predictive relationship. Supporting an interactive effect, Lee (1999) found that reading comprehension was lowest for students with both elevated trait and test anxiety relative to those high in only one anxiety type. Consequently, future research must incorporate measurements of test anxiety, which appears to interact with traits to influence reading achievement. The instability of predictions based solely on trait anxiety levels highlights test anxiety as a missing piece of this phenomenon. Processing efficiency theory, grounded in working memory and attentional mechanisms, provides a cogent rationale for considering trait and test anxiety synergistic influences.

### High-functioning and typical-functioning SLD

Furthermore, it is crucial to differentiate between “high-functioning SLD” and “typical-functioning SLD.” We use typical-functioning SLD here to denote students who continue to show moderate-to-high levels of self-reported literacy challenges, typical of the broader SLD population, rather than implying “typical development.” In contrast, those categorized as high-functioning SLD have fewer self-reported reading and writing difficulties, suggesting that they may have developed more effective compensatory strategies. An increasing body of research has examined both alphabetic languages and Chinese in these age groups, revealing that some individuals with SLD have managed to improve their literacy skills to the extent that they can be classified as having high-functioning SLD (e.g., Birch & Chase, 2004; Chung et al., 2018; Law et al., 2018).

In the present study, we classified high-functioning and typical-functioning SLD students based on their current levels of self-reported literacy difficulties, as measured by the Hong Kong Reading and Writing Behaviour Checklist for Adults. While some studies use longitudinal data to define high-functioning SLD (e.g., Birch & Chase, 2004), our cross-sectional approach aligns with research that utilizes self-report measures to assess functioning levels (e.g., Law et al., 2018). This method allows us to capture the current functional status of students, which is relevant for understanding their immediate academic experiences.

Evidence suggests that individuals with high-functioning SLD may perform similarly to their typically developing peers in certain aspects of reading and writing. However, they may still exhibit difficulties in other areas of literacy (e.g., Deacon et al., 2006; Kemp et al., 2009; Law et al., 2018; Meyler & Breznitz, 2005). Notably, the most definitive findings have emerged from studies exploring the relationships between cognitive abilities, reading-related skills, and reading performance in individuals with high-functioning SLD. For example, Deacon et al. (2006) found that morphological awareness predicted reading comprehension differently in adults with SLD compared to typically developing adults, suggesting unique cognitive processing patterns in the SLD population.

A growing research area examines evaluative anxiety tendencies across student populations, including typically developing students, those with high-functioning SLD, and those with typical-functioning SLD. While both SLD subgroups report higher general trait anxiety than typically developing peers, studies indicate testing-specific anxiety is often amplified uniquely among high-functioning SLD cohorts who otherwise exhibit anxiety matching typically developing levels (Nelson & Harwood, 2011; Peleg, 2009). For example, Nelson and Harwood (2011) found that high school students with mild SLD reported equivalent trait anxiety to typically developing students with comparable low achievement histories. However, the high-functioning SLD group reported substantially greater distress and worry specifically regarding academic testing events relative to their typically developing peers.

Such findings align with theories highlighting cognitive control challenges during assessment scenarios for students with high-functioning information processing deficiencies. Attentional control theory posits that the extensive effort required to compensate for SLD-related difficulties leaves fewer working memory resources to devote toward managing stress appraisals and reactions elicited during evaluations (Eysenck et al., 2007). Consequently, exams degrade attention control efficiency enough to elevate anxiety despite otherwise adequate compensation. Alternatively, the skill demonstration uncertainties amplified by tests may preemptively disrupt processing efficiency for students prone to self-concept doubts, like those with LDs (Eysenck & Calvo, 1992).

Integrating cognitive and social-motivational processes, assessments likely compound uncertainties around competence and skill representations among high-functioning SLD students in particular. Supporting this notion, Peleg (2009) found that evaluations were interpreted as significantly more threatening by high-functioning adolescents with SLD (in the field of reading difficulties) relative to their typical-functioning SLD and typically developing peers. Thus, testing events may levy distress upon a subset of students who maintain average capacities yet lack mastery in demonstrating their atypical skills efficiently.

### The present study

The present study aims to (1) compare trait anxiety and test anxiety among Chinese undergraduates with typical development, high-functioning SLD, and typical-functioning SLD, and (2) examine the mediating role of test anxiety in the relationship between trait anxiety and academic performance across these three groups.

By investigating these relationships in the context of established cognitive theories, the study seeks to contribute a more comprehensive understanding of the factors influencing academic performance in diverse learner populations, particularly those with SLD. The findings may inform the development of theoretically grounded interventions and support strategies tailored to the specific needs of students with SLD, considering their anxiety profiles and cognitive characteristics. Ultimately, this research aims to promote the academic success and overall well-being of Chinese undergraduates with and without different types of SLD by shedding light on the complex interplay between emotional and cognitive factors that shape their educational experiences.

## Methods

### Participants

Our study included a total of 239 s-year undergraduate students from a mix of two public and three private universities located in the central and southern regions of Taiwan. These students came from a variety of academic fields including social sciences, engineering, nursing, and transportation. All participants were monolingual with Chinese as their native language prior to entering the education system and were not specifically chosen based on their enrollment in any particular courses or programs. Out of these participants, 105 were officially recognized as having SLD in Taiwan, with a gender breakdown of 32 males and the rest females.

In the context of Taiwan, students known to have SLD histories were encouraged to reaffirm their status upon entering university. This reidentification is overseen by a special committee designated by the government, which includes educators and psychologists among others. The main aim is to confirm if the students have a documented history of SLD at the secondary education level and to assess if their condition significantly impacts their learning. For new cases seeking identification at the university level, a more rigorous evaluation is mandated, incorporating detailed psychological and linguistic assessments to accurately diagnose learning challenges, such as the Wechsler Adult Intelligence Scale-IV for cognitive abilities (compulsory) and 2019 Reading Comprehension Measurement or reading performance observation for basic reading comprehension and fluency capability (optional), mirroring the process used at earlier educational stages. The reaffirmation of SLD status upon university entry ensures that students continue to receive necessary accommodations. This process is relevant to our study as it confirms the current presence of SLD-related challenges, which may influence anxiety levels and academic performance.

Additionally, 134 undergraduate students without SLD (54 males and the rest female) were also part of the study. These students were carefully selected to align with the SLD group in terms of gender, academic performance, field of study, and university affiliation. Typically developing students were recruited through classroom announcements and flyers across the same universities attended by the SLD participants. We used convenience sampling to select students who matched the SLD groups in terms of age, gender, and academic major. This method was chosen to ensure that the comparison group was demographically similar, minimizing potential confounding variables. Specifically, 105 of these students were directly matched to the SLD group by gender, and the gender balance for the remaining 29 was maintained as closely as possible to equal. These typically developing students were recruited through their SLD-identified peers, ensuring at least one participant from each institution involved in the study was included. None of these students had any record of special education needs or neurological disorders.

### Measures

In this study, assessments were conducted either on an individual basis or in small groups. The self-report questionnaires were presented in Traditional Chinese to match the native language of the participants. Participants were encouraged to read the questions independently, seeking clarification only if needed. Given the anticipation that students with SLD might struggle with independent reading and potentially misinterpret the content, these students were given the questionnaires in more personalized settings, either individually or in pairs, with the examiner reading the questions aloud if the participants requested to do so to facilitate their reading comprehension. This approach ensured that both students with SLD and their peers without reading challenges could fully comprehend the questions. Among the SLD group, 39 individuals utilized this auditory support. Prior to the study, informed consent was secured from all participants.

### Assessment of literacy difficulties

We selected “The Hong Kong Reading and Writing Behaviour Checklist for Adults” to assess learning challenges in participants, which was modified from the work by Ho et al. (2007) to serve as the index of different types of SLD. The checklist focuses on reading and writing challenges, which are core aspects of SLD relevant to academic performance in university settings. All participants, including those without SLD, completed this checklist to assess self-reported reading and writing difficulties. This allowed us to confirm that the typically developing group did not exhibit significant literacy challenges.

The checklist is divided into two sections aimed at identifying the key issues related to SLD, specifically focusing on reading (*n* = 5 items) and writing difficulties (*n* = 7 items), as outlined by Shaywitz (1998). While SLD can also involve mathematics (dyscalculia), the present study concentrated on literacy difficulties, which are often the most frequently reported SLD challenges (Ho et al., 2007). Before implementing this scale, it was confirmed to be suitable for Taiwanese students usage. The instrument comprised a total of 12 questions, and all were scored directly without any items being reverse-scored. Both sections were Likert 5-point scale, so the range of composite scores is from 12 to 60. In our sample, the reliability of the questionnaire, measured by Cronbach’s alpha, was 0.82 for the reading difficulties segment and 0.90 for the writing difficulties segment.

#### Academic performance

In the current study, we employed the first-year university GPAs of the students as a marker of their academic achievement rather than scores from the reading checklist because we aimed to capture a global indicator of students’ success across diverse university majors. This approach draws inspiration from previous research that focused on students at comparable stages of their education, such as the work by Goroshit and Hen (2021). The rationale behind this choice is attributed to the wide range of majors the participants are pursuing at the university level. This diversity renders the application of specialized criteria for assessing academic performance not only challenging but also potentially inaccurate. Academic performance was measured using the cumulative GPA of the participants for their first two semesters. With participants'permission, GPA data were obtained directly from university records, ensuring accuracy and consistency.

#### Trait anxiety

In this study, the trait anxiety component of the State-Trait Anxiety Inventory, a tool crafted by Spielberger in 1983, for measuring overall anxiety levels in individuals was administrated. This component includes twenty questions rated on a four-point Likert scale, where a higher tally suggests increased levels of trait anxiety. The Chinese adaptation of this inventory, verified by Wang and Chung (2016), demonstrated strong convergent validity with a score of 0.90. The reliability of the inventory in our research, as indicated by a Cronbach’s alpha coefficient, was recorded at 0.91, underscoring the consistency of our measurements. Based on our sample, the Cronbach’s alpha coefficient was 0.82.

#### Test anxiety

In this study, the part of test anxiety of School Motivation and Learning Strategies Inventory for College was utilized to evaluate participants’ test anxiety levels, drawing from the adaptation by Stroud and Reynolds (2006). In alignment with the specific objectives of our research, we focused solely on the segment related to test anxiety. This section encompasses 25 questions, among which two are scored in reverse, rated on a five-point Likert scale, where a higher score indicates higher levels of test anxiety. For the individuals participating in this study, the reliability of this segment, as measured by Cronbach’s alpha, was established at 0.88.

### Data analysis

Several statistical methods were used to examine the research aims of the present study. Before that, to differentiate between high-functioning and typical-functioning SLD students, we used the composite scores from the Hong Kong Reading and Writing Behaviour Checklist for Adults. Students with SLD scoring below the mean were classified as high-functioning SLD, indicating fewer self-reported literacy difficulties, while those scoring at or above the mean were classified as typical-functioning SLD. This approach aligns with previous research (e.g., Deacon et al., 2006; Law et al., 2018) that utilized self-report measures to distinguish levels of functioning within SLD populations. The KMO measure of sampling adequacy was 0.50, which is acceptable for factor analysis (Kaiser, 1974), and a Bartlett’s test of sphericity *p* value below 0.001 [*χ*^2^(1) = 182.45, *p* < 0.001], demonstrating the appropriateness of factor analysis for these two dimensions. The analysis revealed that a single factor accounted for 87.0% of the total variance, indicating that this factor significantly captures the variance in literacy difficulties.

Based on the mean literacy difficulty scores, the composite score for literacy difficulties was 33. Students scoring below 33 were classified as high-functioning SLD, and those scoring 33 or above were classified as typical-functioning SLD. Consequently, our analysis focused on three distinct groups: (1) 134 students with typical development; (2) 54 students with typical-functioning SLD; and (3) 51 students with high-functioning SLD.

To address the first research aim, multiple ANCOVAs controlling for age and gender were used to compare the differences between Chinese undergraduates with typical developments and with high- and typical-functioning SLD in trait anxiety and test anxiety. ANCOVA is a common approach to test the differences among groups while taking relevant factors into consideration.

Additionally, to address the second research aim, several statistical methods were used. Firstly, before going for the second research aim, it is crucial to test if different groups of participants showed distinct patterns of relationships between trait anxiety and academic performance. Thus, we conducted a moderation analysis using Hayes'PROCESS macro (model 1) to examine whether the scores of literacy difficulties, which were used to classify two groups of SLD, moderated the relationship between trait anxiety and academic performance. Trait anxiety was the independent variable, academic performance was the dependent variable, and the literacy difficulties score was the moderator. Age and gender were included as covariates. Furthermore, partial correlation analyses, and mediation analyses were used to test the relationships among test anxiety, trait anxiety, and academic performance among Chinese undergraduates with and without different types of SLD. Both mediation analysis and partial correlation analysis were widely used to examine variables’ relationships while considering the impact of relevant factors. Notably, the limited sample size per group in this study, especially for the two SLD groups, might have impeded the statistical power of the mediation analyses for the second research aim (Bearden et al., 1982). We thus used the PROCESS macro, developed by Hayes and Preacher (2014), to examine this hypothesis, utilizing bootstrap procedures recommended for analyzing indirect effects in small samples. This approach has been widely adopted in psychological research (e.g., Hammond et al., 2012; Wang et al., 2018, 2022), with the significance of the results determined by the absence of zero within the resulting confidence interval.

## Results

Before examining the research aims, we implemented several analyses to test the differences in demographic information of the three groups.

Chi-square tests showed no significant association between group membership and field of study, *χ*^2^(4, *N* = 239) = 3.21, *p* > 0.05. An ANCOVA revealed significant differences in GPA among the groups, *F*(2, 234) = 6.78, *p* < 0.01, partial *η*^2^ = 0.06. Furthermore, the age distribution was comparable across the groups [*F* (2, 236) = 1.52, *p* > 0.05; the typical development: *M* = 18.60, *SD* = 1.52; the typical-functioning SLD: *M* = 18.39, *SD* = 0.81; the high-functioning SLD: *M* = 18.28, *SD* = 0.49] which clearly indicated they were similar in age. Also, the gender distribution across the groups yielded a non-significant difference [*χ*^2^(2, *n* = 239) = 4.10, *p* > 0.05; the typical development: male = 53 (39.55%), female = 81 (60.44%); the typical-functioning SLD: male = 13 (24.07%), female = 41 (75.59%); the high-functioning SLD: male = 19 (37.25%), female = 32 (62.75%)]. Finally, the high-functioning SLD group had a higher mean GPA (*M* = 3.35, *SD* = 0.30) compared to the typical-functioning SLD group (*M* = 2.85, *SD* = 0.40), *t*(103) = 7.25, *p* < 0.001, Cohen’s *d* = 1.41. There was no significant difference between the high-functioning SLD group and the non-SLD group (*M* = 3.30, *SD* = 0.35), *t*(183) = 0.93, *p* = 0.35, Cohen’s *d* = 0.15.

The first research aim, which was to examine the differences in trait anxiety and test anxiety among Chinese undergraduates with and without different types of SLD, was addressed by conducting multiple one-way ANCOVAs to compare trait anxiety and test anxiety among Chinese undergraduates with typical development, high-functioning SLD, and typical-functioning SLD, while controlling for age and gender. The results are shown in Table 1.

**Table 1:** Comparisons of the three groups with controlling for age and gender

	1. Typical development		2. Typical-functioning SLD		3. High-functioning SLD		*F* (2, 234)	*η*^2^	Post hoc
	(*n* = 134)		(*n* = 54)		(*n* = 51)				
	*M*	*SD*	*M*	*SD*	*M*	*SD*			
Literacy difficulties	24.20	6.57	38.96	6.26	22.24	5.13	103.64**	0.49	1 = 3 > 2
Trait anxiety	55.68	11.50	56.87	12.40	49.51	14.31	5.86**	0.05	1 = 2 > 3
Test anxiety	72.76	12.24	77.26	13.17	66.59	13.63	8.92**	0.07	2 > 1 > 3

^**^p <.01

Firstly, an ANCOVA revealed significant differences in literacy difficulties among the three groups with controlling for age and gender, *F*(2, 234) = 103.64, *p* < 0.001, partial* η*^2^ = 0.49. Post hoc tests showed that both the typical development group and the high-functioning SLD group reported significantly fewer difficulties compared to the typical-functioning SLD group (*M*_*diff*_ = − 14.76 and − 16.72, *SE* = 1.05 and 1.26, *p* < 0.01 for both).

For trait anxiety, the ANCOVA, controlling for age and gender, revealed significant differences among the three groups, *F*(2, 234) = 5.86, *p* < 0.01, *η*^2^ = 0.05. Post hoc comparisons using Bonferroni correction indicated that the high-functioning SLD group reported significantly lower levels of trait anxiety compared to both the typical development group (*M*_*diff*_ = 6.48, *SE* = 2.08, *p* < 0.01) and the typical-functioning SLD group (*M*_*diff*_ = 7.23, *SE* = 2.43, *p* < 0.01). No significant difference was found between the typically developing and the typical-functioning SLD groups (*M*_*diff*_ = 0.75, *SE* = 2.09, *p* > 0.05).

Regarding test anxiety, the ANCOVA also yielded significant differences among the three groups, *F*(2, 234) = 8.92, *p* < 0.01, *η*^2^ = 0.07. Post hoc comparisons with Bonferroni correction revealed that the high-functioning SLD reported significantly lower levels of test anxiety compared to both the typically developing group (*M*_*diff*_ = 6.05, *SE* = 2.15, *p* < 0.01) and the typical-functioning SLD group (*M*_*diff*_ = 10.52, *SE* = 2.51, *p* < 0.01). Furthermore, the typically developing group reported significantly lower levels of test anxiety than the typical-functioning SLD group (*M*_*diff*_ = 4.47, *SE* = 2.16, *p* < 0.01).

The second research aim, which was to test the mediating role of test anxiety in the relationship between trait anxiety and academic performance, was addressed by conducting partial correlation analyses, with controlling for age and gender, and mediation analyses to examine the relationships among trait anxiety, test anxiety, and academic performance in the three groups. Due to the limited sample size per group, a bootstrapping technique with a subsample of 5000 was employed to test the relationships among the variables.

As mentioned above, one moderation analysis was implemented to examine if different groups of participants showed distinct patterns of relationships between trait anxiety and academic performance. Overall, the regression model was significant, *R*^2^ = 0.27, *F*(4, 234) = 22.19, *p* < 0.001. Crucially, the interaction term (trait anxiety × literacy difficulties) was also significant, *β* = –0.15, *SE* = 0.05, *t* = –3.00, *p* < 0.01, 95% *CI* [− 0.25, − 0.05]. Simple slope analyses indicated that for students with higher levels of literacy difficulties, trait anxiety had a significantly stronger negative association with academic performance (*β* = − 0.40, *SE* = 0.10, *t* = − 4.00, *p* < 0.001). Conversely, among students with lower levels of literacy difficulties, the relationship between trait anxiety and academic performance was smaller and not statistically significant (*β* = − 0.07, *SE* = 0.08, *t* = − 0.88, *p* = 0.38). These findings suggest that higher literacy difficulties intensify the negative impact of trait anxiety on academic performance.

**Fig. 1:**
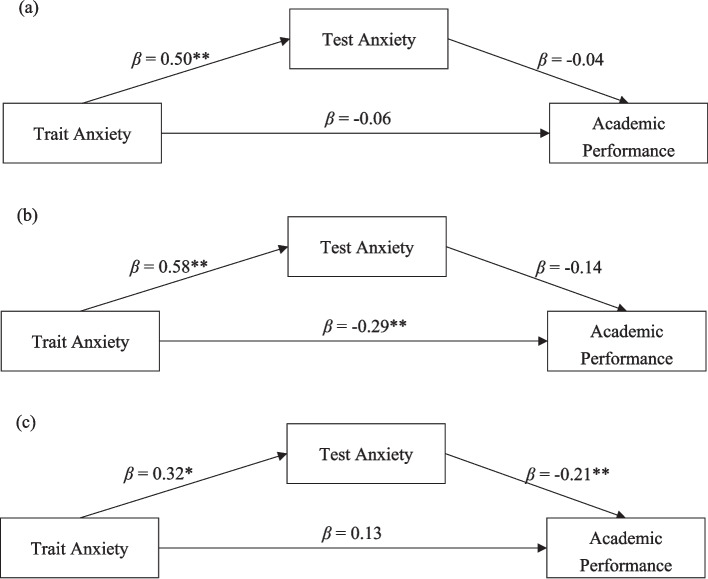
The direct and indirect effects of trait anxiety to academic performance through test anxiety of three groups. **a** Typical development. **b** Typical-functioning SLD. **c** High-functioning SLD. *p <.05, **p <.01

In the typically developing group, partial correlations revealed nonsignificant associations between trait anxiety and academic performance (*r* = − 0.14, *p* > 0.05) and between test anxiety and academic performance (*r* = − 0.16, *p* > 0.05). The mediation analysis showed, as Fig. 1a, nonsignificant effects for both direct effect of trait anxiety on academic performance (*β* = − 0.05, *t* = − 1.14, *p* > 0.05) and indirect effect through test anxiety (*β* = − 0.02, *BootLLCI* = − 0.0875, *BootULCI* = 0.0331).

For the typical-functioning SLD group, we found partial correlations indicating significant associations between academic performance and test anxiety (*r* = − 0.38, *p* < 0.01) but not trait anxiety (*r* = 0.09, *p* > 0.05). The mediation analyses, as Fig. 1b, revealed significant direct effects of trait anxiety on academic performance (*β* = − 0.28, *t* = − 3.14, *p* < 0.01) but not the indirect effect through test anxiety (*β* = − 0.08, *BootLLCI* = − 0.2272, *BootULCI* = 0.0364).

On the contrary, we found a significant association with academic performance was only with trait anxiety (*r* = − 0.34, *p* < 0.01) and not test anxiety (*r* = 0.11, *p* > 0.05) for the high-functioning SLD group. The mediation analyses, as Fig. 1c, revealed nonsignificant direct effects of trait anxiety on academic performance (*β* = 0.12, *t* = 1.65, *p* > 0.05) but a significant indirect effect through test anxiety (*β* = − 0.07, *BootLLCI* = − 0.1834, *BootULCI* = − 0.0017).

In summary, the results indicate that Chinese undergraduates with high-functioning SLD report lower levels of trait anxiety and test anxiety compared to their typically developing peers and those with typical-functioning SLD. Furthermore, the relationship between trait anxiety and academic performance is mediated by test anxiety in the high-functioning SLD group, but not in either the typically developing group or the typical-functioning SLD group, which found only a direct effect of trait anxiety on academic performance.

## Discussion

The present study aimed to investigate the differences in trait anxiety and test anxiety among Chinese undergraduates with typical development, high-functioning SLD, and typical-functioning SLD, and to examine the mediating role of test anxiety in the relationship between trait anxiety and academic performance across these three groups. The findings provide valuable insights into the complex interplay between anxiety and academic performance in diverse learner populations, particularly those with SLD. Our grouping method, based on self-reported literacy difficulties, is consistent with studies that recognize the variability within the SLD population (e.g., Chung et al., 2018). By specifying our cutoff scores, we enhance the comparability of our work with existing literature. Also, the comparable GPAs between the high-functioning SLD and non-SLD groups, as shown above, suggest that the high-functioning SLD students are performing academically at a similar level to their peers, which may explain their lower levels of test anxiety. The lack of significant differences in majors indicates that the choice of field does not account for the observed differences.

### Differences in trait anxiety and test anxiety

The results revealed significant differences in both trait anxiety and test anxiety among the three groups. Surprisingly, the high-functioning SLD group reported significantly lower levels of trait anxiety compared to both the typically developing group and the typical-functioning SLD group. This finding contradicts previous research that has consistently shown higher levels of trait anxiety in individuals with SLD compared to their typically developing peers (Nelson & Harwood, 2011; Peleg, 2009). One possible explanation for this discrepancy is that high-functioning SLD students may have developed effective coping strategies or received targeted support that has helped them manage their anxiety. Additionally, their higher academic performance may contribute to a more positive self-perception and lower anxiety levels. Also, another explanation is that earlier studies documenting elevated trait anxiety in students with SLD (e.g., Peleg, 2009) focused on adolescents or younger populations who may not have developed effective compensatory strategies yet.

Regarding test anxiety, the high-functioning SLD group also reported significantly lower levels compared to both the typically developing group and the typical-functioning SLD group. Furthermore, the typical-functioning SLD group exhibited the highest levels of test anxiety among the three groups. These findings are consistent with previous research that has identified test anxiety as a significant challenge for students with SLD (Nelson et al., 2015; Whitaker-Sena et al., 2007). The current study extends these findings by demonstrating the differential experiences of test anxiety among high-functioning and typical-functioning SLD subgroups.

### Mediating role of test anxiety

Beforehand, we found a significant moderation effect that suggests that literacy difficulties exacerbate the negative impact of trait anxiety on academic performance. This finding supports the notion that students with more significant literacy challenges are more vulnerable to the detrimental effects of anxiety. It provides a rationale for testing the mediation analyses separately for the three groups in this study. Furthermore, the mediation analyses revealed distinct patterns in the relationship between trait anxiety, test anxiety, and academic performance across the three groups. In the typically developing group, neither direct nor indirect effects of trait anxiety on academic performance were significant. This finding is inconsistent with some previous studies that have shown a direct relationship between trait anxiety and academic performance in non-SLD populations (Lee, 1999; Tysinger et al., 2010). The discrepancy between our findings and previous studies may be due to cultural differences in the expression of anxiety or the specific characteristics of our sample. It's also possible that other unmeasured factors, such as coping strategies or social support, play a role. Further research is needed to explore these possibilities and determine the generalizability of our results.

In the typical-functioning SLD group, trait anxiety had a significant direct effect on reading performance, but test anxiety did not serve as a mediator. This finding suggests that for typical-functioning SLD students, trait anxiety may directly impact their academic performance without the need for test anxiety as an intermediary. This result is somewhat inconsistent with previous research that has identified test anxiety as a significant mediator in the relationship between trait anxiety and academic performance in students with SLD (Grills-Tacquechel et al., 2012; Nelson & Harwood, 2011). However, the current study’s focus on typical-functioning SLD students may account for this discrepancy, as these students may have developed adaptive coping strategies that mitigate the impact of test anxiety on their reading performance.

In the high-functioning SLD group, test anxiety significantly mediated the relationship between trait anxiety and reading performance, while the direct effect of trait anxiety on academic performance was not significant. This finding aligns with the cognitive-interference model (Sarason, 1984) and the processing efficiency theory (Eysenck & Calvo, 1992), which propose that anxiety can impair cognitive performance by consuming working memory resources and disrupting attentional control. The results are also consistent with previous research that has identified test anxiety as a significant mediator in the relationship between trait anxiety and academic performance in students with SLD (Grills-Tacquechel et al., 2012; Nelson & Harwood, 2011).

The differential patterns of mediation observed in the high-functioning and typical-functioning SLD groups may be rooted in their distinct cognitive profiles and compensatory mechanisms. High-functioning SLD students, by virtue of their better academic performance and potentially more effective coping strategies, may have developed a greater capacity to mitigate the direct impact of trait anxiety on their academic performance (Swanson & Hsieh, 2009; Trainin & Swanson, 2005). However, their vulnerability to test anxiety and its indirect effect on academic performance may be a result of the specific cognitive demands and attentional disruptions triggered by evaluative situations (Eysenck et al., 2007; Sarason, 1984).

Previous research has suggested that high-functioning individuals with SLD may possess unique cognitive strengths and compensatory strategies that allow them to achieve academic success despite their learning difficulties (Cavioni et al., 2020; Firth et al., 2013; MacCullagh et al., 2017). These compensatory mechanisms may include enhanced problem-solving skills, adaptability, and the ability to allocate cognitive resources efficiently in non-evaluative contexts (Cortiella & Horowitz, 2014; Heiman & Precel, 2003). Such strengths may help to explain why high-functioning SLD students in the present study demonstrated lower levels of trait anxiety and a reduced direct impact of trait anxiety on their reading performance. While our data suggest that high-functioning SLD students may have developed coping strategies, we acknowledge that we did not directly measure compensatory mechanisms. Therefore, interpretations regarding compensation should be considered tentative. Similarly, we must consider that some non-SLD students might employ strategies to manage undiagnosed literacy difficulties.

Additionally, the cognitive demands and attentional disruptions associated with evaluative situations may pose a unique challenge for high-functioning SLD students, rendering them more susceptible to the negative effects of test anxiety on their academic performance (Eysenck et al., 2007; Sarason, 1984). The heightened performance pressure and the need to allocate cognitive resources efficiently in test-taking contexts may strain the compensatory mechanisms that typically support their academic functioning (Heiman & Precel, 2003; Swanson & Hsieh, 2009). Consequently, the indirect effect of trait anxiety on academic performance through test anxiety may be more pronounced in this subgroup.

In contrast, typical-functioning SLD students, who may not have developed the same level of compensatory strategies and cognitive strengths as their high-functioning counterparts (Cavioni et al., 2020; Firth et al., 2013), may be more directly impacted by trait anxiety across both evaluative and non-evaluative contexts. The absence of a mediating effect of test anxiety in this subgroup suggests that their academic performance may be more consistently influenced by their overall anxiety levels, rather than being specifically triggered by the cognitive demands of test-taking situations (Nelson & Harwood, 2011; Whitaker-Sena et al., 2007).

### Limitations and future directions

While this study contributes to our understanding of the relationships between anxiety and academic performance in Chinese undergraduates with and without different types of SLD, several limitations should be acknowledged. The sample size of the high-functioning and typical-functioning SLD groups was relatively small, which may have limited the statistical power of the analyses. Future research should aim to replicate these findings with larger sample sizes to ensure the robustness of the results. Additionally, the study relied on self-report measures of anxiety and reading difficulties, which may be subject to response bias. Future research could incorporate objective measures of academic performance and physiological indicators of anxiety to provide a more comprehensive assessment of these constructs. The cross-sectional design of the study precludes causal inferences about the relationships between anxiety and reading performance. Longitudinal studies are needed to examine the developmental trajectories of anxiety and reading difficulties in students with SLD and to identify potential bidirectional relationships between these variables. Finally, although we included self-reported literacy difficulties from the non-SLD group, it is possible that some individuals with undiagnosed SLD were present. Future studies should include formal assessments to rule out unrecognized cases.

## Conclusion

Despite the limitations, the findings of this study have important implications for educators and mental health professionals working with Chinese undergraduates with and without different types of SLD. The results underscore the need for targeted interventions and support strategies that address the specific anxiety profiles and cognitive characteristics of students with SLD, taking into account their level of academic functioning. For typical-functioning SLD students, interventions focusing on maintaining their adaptive coping strategies and fostering a positive self-perception may be particularly beneficial, as trait anxiety appears to have a direct impact on their reading performance. For high-functioning SLD students, interventions aimed at reducing test anxiety may be more appropriate, as test anxiety serves as a significant mediator in the relationship between trait anxiety and reading performance. Distinguishing between typical-functioning and high-functioning SLD is important for tailoring educational interventions. While these classifications may not be easily used in practice formally, recognizing the spectrum of functioning within SLD can help educators and clinicians develop individualized support strategies. Similarly, differentiating between trait and test anxiety allows for targeted interventions; for instance, high-functioning SLD students may benefit more from test anxiety reduction techniques. By understanding these nuances, practitioners can better address the specific needs of each student.

The present study sheds light on the complex relationships between trait anxiety, test anxiety, and academic performance in Chinese undergraduates with and without different types of SLD. The findings highlight the distinct anxiety profiles and differential patterns of direct and indirect effects of trait anxiety on academic performance among students with high-functioning SLD, typical-functioning SLD, and typical development. These results have important implications for the development of targeted interventions and support strategies that address the unique needs of these learner populations. By promoting a deeper understanding of the emotional and cognitive factors that shape the educational experiences of students with SLD, this research contributes to the ongoing efforts to foster inclusive and supportive learning environments in higher education.

## Data Availability

Due to the nature of this research, participants of this study did not agree for their data to be shared publicly, so supporting data is not available.
